# Whole Grains Contribute Only a Small Proportion of Dietary Fiber to the U.S. Diet

**DOI:** 10.3390/nu9020153

**Published:** 2017-02-17

**Authors:** Sibylle Kranz, Kevin W. Dodd, Wen Yen Juan, LuAnn K. Johnson, Lisa Jahns

**Affiliations:** 1 Department of Kinesiology, University of Virginia, Charlottesville, VA 22904, USA; 2 National Cancer Institute, Bethesda, MD 20892, USA; doddk@mail.nih.gov; 3 Nutrition Assessment and Evaluation, Office of Nutrition and Food Labeling, Center for Food and Applied Nutrition, United States Food and Drug Administration, College Park, MD 20740, USA; wenyen.juan@fda.hhs.gov; 4 United States Department of Agriculture, Agricultural Research Service, Grand Forks Human Nutrition Research Center, Grand Forks, ND 58203, USA; luann.johnson@ars.usda.gov (L.K.J.); lisa.jahns@ars.usda.gov (L.J.)

**Keywords:** whole grain intake, dietary fiber, nutrition monitoring, Dietary Guidelines for Americans, healthy diet, sources of dietary fiber

## Abstract

Dietary fiber (DF), found in whole fruits, vegetables, and whole grains (WG), is considered a nutrient of concern in the US diet and increased consumption is recommended. The present study was designed to highlight this critical importance of the difference between WG, high-fiber WG, and sources of fiber that are not from WG. The study is based on the two-day diets reported consumed by the nationally representative sample of Americans participating in What We Eat In America, the dietary component of the National Health and Nutrition Examination Survey from 2003–2010. Foods consumed were classified into tertiles of DF and WG and the contribution of fiber by differing levels of WG content were examined. Foods containing high amounts of WG and DF only contributed about 7% of total fiber intake. Overall, grain-based foods contributed 54.5% of all DF consumed. Approximately 39% of DF came from grain foods that contained no WG, rather these foods contained refined grains, which contain only small amounts of DF but are consumed in large quantities. All WG-containing foods combined contributed a total of 15.3% of DF in the American diet. Thus, public health messaging needs to be changed to specifically encourage consumption of WG foods with high levels of DF to address both recommendations.

## 1. Introduction

### 1.1. Dietary Fiber

Dietary fiber (DF), which occurs in whole fruits, vegetables, and whole grains (WG), is considered a nutrient of concern in the US diet and increased consumption is recommended. [1] In the UK, recommendations are 30 g/day for adults [2]. The recommended DF intake in the US is at least 14 g of fiber per 1000 kcal of total energy consumed, which translates to roughly 19 g per day for children 1–3 years old to between 25–35 g per day for adults [3]. Although intake increased slightly among some Americans over a 10-year period [4], fiber intake is low in the American diet with less than 3% of the population meeting recommendations [5,6]; most Americans consume very low amounts [7]. As fiber intake is inversely associated with body weight [8,9,10,11,12], cardiovascular disease (CVD) risk factors [13,14,15], some cancers, and potentially type 2 diabetes and other chronic diseases [16,17], it is important to increase consumption to improve population health. The greatest source of fiber in the diet is vegetables (22%), followed by mixed dishes (12%), yeast breads (12%), and fruits (11%) [4]. 

### 1.2. Whole Grains

One of the sources of dietary fiber, WG, has also been associated with decreased risks of obesity, type 2 diabetes, and CVD [18,19,20]. The World Health Organization recommends consuming WG as part of a healthy diet [21]. Many countries and organizations also have recommendations for WG intake, although intake guidelines vary substantially [22]. WG were first introduced as a food group for recommended intake in the Dietary Guidelines for Americans (DGA) 2005 and have been maintained as part of the USDA Healthy US-Style Food Patterns as a food group to be recommended in the 2015–2020 DGA [23,24]. The 2015–2020 DGA provides food-based guidance and recommends that Americans consume at least one-half of their daily suggested total grains as WG, which is equivalent to at least 3 oz. equivalents per day for many individuals consuming more than 1600 calories per day. However, nationally representative intake data suggest that only 2%–7% of Americans meet this recommendation [25,26]. The greatest sources of WG in the diet of adults are yeast breads (27%), ready-to-eat (RTE) cereals (23%), and pastas, cooked cereal and rice (21%) [4]. However, those food sources are not generally 100% WG. The American diet does not include many foods that are 100% WG, but rather foods that contain some small proportion of WG, such as breads, cereals, or pastry made with some WG flour. The US Food and Drug Administration classification of WG is limited to those products which retain all of the main biological components of the grain (germ, endosperm, and bran) in the same relative proportion as in the intact grain [27], which is not necessary related to the amount of dietary fiber. Therefore, the “whole grain”-containing foods recommended by the DGA encompass a wide variety of foods, ranging from those with proportionally low to those with very high amounts of dietary fiber, depending upon the type of grain.

### 1.3. Significance

DGA recommendations focus on increasing the consumption of WG [28,29] to half of all consumed grains, however, due to the large variation of dietary fiber content in WG foods (g/100 g), it is important to differentiate between high fiber and low fiber WG foods. The present study was designed to highlight this critical importance of the difference between WG, high-fiber WG, and sources of fiber that are not from WG. A previous analysis using the 2009–2010 National Health and Nutrition Examination Survey (NHANES) found that fiber consumption was related to WG consumption [30]; however, the sample was small (only one NHANES wave), which limited the amounts of food that were included in the analysis. Due to the high number of foods in the US food supply that use the words “whole grain” in several locations on food packaging for marketing purposes, a timely research question is: “what are the food sources of DF consumed by the American population, and what proportion of dietary fiber is contributed by WG?” To answer this question, we pooled data from 8 years of NHANES surveys in the US, from 2003–2004 to 2009–2010 and examined the relationships between WG foods and DF from these foods.

## 2. Materials and Methods

### 2.1. Data Set

The study is based on the two-day diets reported consumed by the nationally representative sample of Americans participating in What We Eat In America (WWEIA), the dietary component of NHANES, and is reflected in oz. equivalent of the MyPlate “whole grains” food group [31]. NHANES is a nationally representative, cross-sectional survey of the non-institutionalized civilian U.S. population and is conducted by the National Center for Health Statistics, Centers for Diseases Control and Prevention. Details of both WWEIA and NHANES can be found elsewhere [32]. In short, participants completed two interviewer-assisted 24-h recalls; day one recalls were administered in-person and day two recalls were conducted over the phone. Primary caregivers reported proxy intake for children less than six years old and assisted children ages 6–11 years; individuals age 12 and older self-reported their previous day’s food intake. All dietary interviews were conducted by trained interviewers using the U.S. Department of Agriculture’s Automated Multiple-Pass Method [33].

For this study, total grain, WG, and DF intakes were estimated using day one dietary intake data collected from 34,391 individuals aged 2 years and older participating in the WWEIA, NHANES 2003–2004, 2005–2006, 2007–2008, and 2009–2010 continuing surveys.

WG were defined as grains that include the entire grain kernel—the bran, germ, and endosperm. WG values were obtained for all reported foods in all survey years using the MyPyramid Equivalents Database (MPED), 2.0 for USDA Survey Food Codes, 2003–2004 (MPED 2.0) and the Center for Nutrition Policy and Promotion Addendum to MPED 2.0 [34,35]. At the time of analysis, the new MPED including the Equivalents database for NHANES 2009–2010 was not available, thus the authors generated a proxy food list to identify the food codes containing WG. Since only a small proportion of Americans meet the WG or the DF dietary guidance, consumption patterns were not based on the intake level of the individuals but conducted on the food level, to help explain why so many Americans have insufficient amounts of WG and DF in their diets. Foods containing WG were categorized as described below. Dietary fiber intake values were obtained from the USDA Food and Nutrition Database for Dietary Studies (FNDDS) version 2.0 and version 4.1 [36].

### 2.2. Study Variables

Four categories of dietary fiber density food (no, low, medium, or high dietary fiber) and five categories of WG food (not a grain food, a grain food with no WG, a grain food with low amounts of WG, a grain food with medium amounts of WG, and a grain food with high amounts of WG) were established. The dietary fiber and WG categories “low, medium, and high” were established based on unweighted tertiles of dietary fiber density (g/100 g of food) and of WG density (oz. equivalents /100 g of food) of all food codes in the FNDDS that were reported consumed at least once in day one dietary intake data. As this research focuses on WG and fiber intake, foods not containing grains were not included in analysis beyond the description of their contribution to total fiber intake.

To maintain the nationally representative character of the data, the calculation of the proportion of foods consumed in each of the fiber (low, medium, high) and WG (no grains, no WG, low WG, medium WG, and high WG) categories in the total population and the male and female population of 2–18 years old and 19–85 years old were computed using survey sample weights. All analyses were performed using SAS, version 9.3 (SAS Institute, Cary, NC, USA). It is important to point out that this analysis was conducted to identify the foods available to the consumer based on their WG and DF content and their contribution to average usual intake, not to any particular individual’s dietary intake. This approach was chosen to highlight the complexity of access to high-fiber foods in the US food supply.

## 3. Results

The number of survey respondents and their socio-economic characteristics are reflected in Table 1. Approximately 50% of the sample were males, more individuals were Non-Hispanic white and more had higher than high school education, which is reflective of the US census data. The educational levels of individuals 18 years and younger are not reported here, as they are likely still in school and their terminal degrees are not known. A greater proportion of younger individuals was from low-income families while the majority of the adult population was from medium or high income households.

All foods analyzed were reported as consumed by the population. As shown in Table 2, foods containing high amounts of WG and DF only contributed about 7% of total fiber intake. Overall, all grain-based foods consumed only contributed 54.5% of all DF consumed—the remaining 45.5% of DF was supplied from non-grain foods. Approximately 39% of DF came from grain foods that contained no WG, rather these foods contained refined grains, which contain only small amounts of DF but are consumed in large quantities. All WG-containing foods combined contributed a total of 15.3% of DF in the American diet.

**Figure 1:**
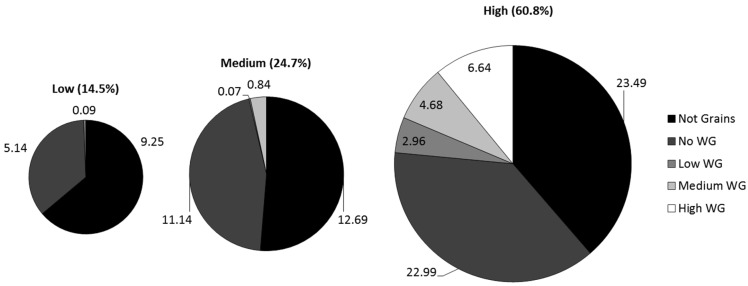
Percent of total dietary fiber intake provided by low, medium, and high fiber foods (defined by g fiber/100 g food) in the diets of Americans ages 2–85 years old.

The distribution of total DF contributed by each of the five WG categories is reflected in Figure 1. The distribution did not differ between the total population and the age and gender subgroups, thus, results from the subgroups are not included but are available upon request. As the pie charts show, low DF consumers obtained approximatly 2/3 of their daily average DF from food sources that were not grains. The proportional contribution of non-grains decreased with increasing level of total DF in the diet, in that the high DF diets were characterized by having only approximately 35% from non-grain food sources but approximately 25% were from medium or high WG foods.

## 4. Discussion

Sixty percent of Americans report trying to consume more fiber and WG; however, 35% believe that they are already getting enough WG [37]. Recent discussions on venues to improve the American diet have concluded that while WG foods are a good source of several essential nutrients and should be continued to be emphasized as part of a healthy diet, efforts to improve DF intake will fail if they are based solely on the recommendation to increase WG foods [38]. Therefore, such efforts should specifically encourage the consumption of high-fiber foods, including high-fiber WG foods such as multigrain bread, popcorn, or high-fiber ready-to-eat cereal.

Results from the present study are in concordance with these findings. Data indicate that the WG foods consumed by Americans contribute very little to total DF intake. Estimates show that more than three-fourths of DF consumed by adults and children were provided by foods that are not grains or are refined grains but do not contain WG. It is noteworthy to point out that the proportion of DF from WG products might be higher if individuals would consume the recommended amount of WG. However, since only a very small proportion of the American population falls into that category, no generalizable models to predict potential DF from WG foods can be established [28,38].

In previous research, we showed that the majority of DF in children’s diets was provided by high consumption of low-fiber foods, and that healthy-weight children were more likely to consume high-fiber foods than overweight/obese children [39]. Thus, in an effort to address overweight/obesity in American children the relationship between WG and DF has to be clarified to motivate the population to seek foods that are high in DF and WG. Currently, this differentiation is not clear, which is due in part to the fact that WG consumption is encouraged by the DGA in terms of “servings of WG-containing foods” while the recommendation for DF expressed in the DRI as 14 g/1000 kcal consumed is not as widely distributed. DF consumers had diets that were disproportionately high in non-grain or non-WG foods; only a very small proportion of the fiber consumed originated from low WG food. In the medium and high DF diets, however, the diversity of the sources of fiber increased and included at least some amounts of medium or high WG foods. One could assume that even low DF consumers would have some portion of the DF they consume from medium or high WG foods but the data indicate individuals with low DF select an intake pattern in which those foods are avoided. Future research is needed to understand (a) if consumers are aware that their diets are low in DF and (b) the factors leading to this pattern of low WG and low DF diets. Once this information has been generated, potential intervention points to move consumers to a diet that contains at least some medium and high WG foods can be developed and implemented.

It is noteworthy to point out that some of the consumer’s misconceptions about their DF intake are likely based on the use of two different metrics: front-of package labeling about the food being a “good” source of WG, while food’s content of DF can be labeled using the CODEX definitions for nutrient claims (“high” in fiber (must contain 6 g DF/100 g of food, or 3 g DF per 100 kcal from the food, or 20% of dietary reference values delivered in one serving). To eliminate this confusion, packages would have to be labeled with two statements: one concerning the level of WG in the product and another to address the amount of DF in the food.

As all studies based on the NHANES, this study too has several limitations. First, our analysis was based on self-reported dietary intake records, which may include biased reporting and may not be reflective of the rapidly changing U.S. food supply. Many more high-fiber foods may be available in the American food supply but if they were not reported by the participants of NHANES, they were not included in this analysis. Our approach, therefore, may well underestimate the number of high WG, high-fiber foods in the marketplace. Also, the food industry has responded to the recommendation to consume more dietary fiber by reformulating food products and adding WG, fiber, or both. Currently it is not possible to differentiate between naturally occurring fibers and fiber added to products, such as wheat bran added to breads. Furthermore, the food supply now also includes different, new, types of fiber, which are found in foods in varying proportions and which may have disparate health effects.

Despite the limitations, the strengths of this study include the large, nationally representative sample and the use of four NHANES waves, which increases the variety and number of foods included in the analysis. Also, unlike other researchers who categorize consumers by their level of WG or DF intake, we focused on a food-based analysis to help explain the high proportion of Americans not meeting the intake guidelines. Although this effort requires the establishing of a different set of cut points, it allows the data-driven analysis, thus enhancing our understanding of the population intake patterns. In this particular instance, the methodology specifically developed for this project to optimally estimate WG and DF intake sources moves the field beyond other studies, which were limited by describing intakes in pre-established groups of people who meet or fail to meet the intake recommendations [40]. Based on the study presented here, public health efforts to change the intake behavior of those who do not meet the WG and DF intake recommendations can be developed.

## 5. Conclusions

The Dietary Guidelines for Americans include the recommendation to consume at least 50% of grains from WG and the DRI stipulate that a healthy diet contain 14 g of DF per 1000 kcal consumed. Most Americans don’t meet either recommendation. The data presented here show that the WG products consumed by Americans are very low in dietary fiber, thus, public health messaging needs to be changed to encourage consumption of WG foods with high levels of DF to address both recommendations.

## Figures and Tables

**Table 1:** Characteristics of study population, 2003–2010.

Participant Characteristic	Children 2–18		Adults 19 & Older	
	**Males**	**Females**	**Males**	**Females**
Unweighted sample size (*N*)	6775	6646	10,181	10,789
Gender (%)	50.7	49.3	47.9	52.1
Race-ethnicity (%)				
Non-Hispanic white	30.9	29.3	34.0	36.8
Non-Hispanic black	7.0	7.4	5.1	6.3
Mexican American	9.5	9.1	6.1	6.1
Other	3.3	3.4	2.6	2.8
Poverty Income Ratio (PIR) (%)				
<1.3	15.5	16.4	9.0	12.0
1.3–1.84	5.8	5.4	4.6	5.8
1.85–3.4	12.8	11.4	11.5	12.5
>3.4	17.1	15.7	22.8	21.8
Education (%)				
No high school diploma	N/A	N/A	8.9	9.8
High school graduate	N/A	N/A	12.3	13.0
More than high school	N/A	N/A	26.6	29.2

**Table 2:** Percent of whole grain and fiber consumed by tertiles of WG (oz. equivalents/100 g food) and dietary fiber (g/100 g food) in diets of Americans ages 2–85 years, 2003–2010.

Whole Grain (Tertiles)	Fiber (Tertiles)	WG %	Fiber %	% Reporting
High WG (WG ≥ 1.33)	High Fiber (DF ≥ 1.7)	50.4	6.6	7.9
Medium WG (0.71 < WG < 1.33)	High Fiber (DF ≥ 1.7)	29.9	4.7	7.9
	Medium Fiber (0.9 < DF < 1.7)	10.5	0.8	1.1
	Low Fiber (0 < DF ≤ 0.9)	0.2	0.01	0.02
Low WG (0 < WG ≤ 0.71)	High Fiber (DF ≥ 1.7)	8.1	3.0	6.8
	Medium Fiber (0.9 < DF < 1.7)	0.1	0.1	0.2
	Low Fiber (0 < DF ≤ 0.9)	0.9	0.1	0.6
No WG	Any Fiber (DF > 0)	0.0	39.2	33.1
Total		100.0	54.5	
